# Clinical presentation and surgical management of perforated peptic ulcer in a tertiary hospital in Mogadishu, Somalia: a 5-year retrospective study

**DOI:** 10.1186/s13017-022-00428-w

**Published:** 2022-05-16

**Authors:** Abdihamid Mohamed Ali, Abdulkadir Nor Mohamed, Yahye Garad Mohamed, Salim İdris Keleşoğlu

**Affiliations:** 1 Department of General Surgery, Mogadishu Somali Turkey Recep Tayyip Erdoğan Training and Research Hospital, Mogadishu, Somalia; 2 Department of Radiology, Mogadishu Somali Turkey Recep Tayyip Erdoğan Training and Research Hospital, Mogadishu, Somalia; 3 Department of General Surgery, İzmir Tepecik Training and Research Hospital, İzmir, Turkey

**Keywords:** Peptic ulcer perforation, Helicobacter pylori, NSAIDs, Exploratory laparotomy

## Abstract

**Background:**

Perforated peptic ulcer is a common surgical emergency condition worldwide, which is associated with significant morbidity and mortality if early diagnosis and immediate surgical management were not carried out. Perforation occurs in roughly 5% of PUD patients during their lifetime; this study aimed to explore the wide range of clinical presentations, associated risk factors, complications, and surgical management of perforated peptic ulcer patients.

**Methods:**

A 5-year retrospective observational study on the clinical presentation and surgical management of perforated peptic ulcer is carried out in a tertiary hospital in Mogadishu, Somalia, Department of General Surgery, from January 2017 to December 2021. We included all patients undergoing operations with an intraoperative confirmed diagnosis of perforated peptic ulcer at the general surgery department. For operated patients, follow-up evaluation was performed in the outpatient department.

**Results:**

Fifty-one patients underwent an emergency operation for perforated peptic ulcer during the study period. The sociodemographic distribution of patients was 45 (88.2%) males and 6 (11.8%) females, giving a male-to-female ratio of 7.5:1. The mean age of patients was 35.5 ± 16.8 years, and the peak frequency was in the third decade. The commonest presenting symptoms were sudden onset of severe epigastric pain in 42 (82.4%) patients. Patients who presented perforated peptic ulcer within 24 h of initiation of symptoms were free from complications. Age-group and delayed presentation > 48 h after onset of symptoms were linked to postoperative complications and were statistically significant (P 0.032 and P 0.005), respectively. Four patients died (mortality rate of 7.8%). Two patients were reoperated because of the failed primary repair, and 4 patients had > 5 cm intra-abdominal abscess image-guided percutaneous drainage, and the rest were given antibiotic therapy according to peritoneal fluid culture and sensitivity results. The most common microorganism isolated was *E. coli* 22% and Klebsiella 11%. Other rare microorganisms (pseudomonas, Staphylococcus aureus, and Candida spp.) were identified. In half (51%) of the patients with peritoneal fluid culture, no microorganism growth was seen.

**Conclusion:**

The distribution of perforated peptic ulcer is common in the young age-group in the third decades of life. Delayed presentation of the disease is linked because most patients arrived from remote areas where proper facilities of health care and health education are not available and the patient might come to the hospital in an advanced stage of the disease. We suggest conducting further researches, health awareness related to complications over-the-counter drugs self-medication, and bad habit including smoking, and to improve health-seeking behaviors of society.

## Background

Peptic ulcer disease (PUD) affects 4 million people globally each year. The incidence of PUD has been estimated to be between 1.5 and 3%. Perforation occurs in roughly 5% of PUD patients during their lifetime. Perforated peptic ulcer is a common surgical emergency condition worldwide, which is associated with significant morbidity and mortality if early diagnosis and immediate surgical management were not carried out, having a mortality rate that ranges from 1.3 to 20% [1].

PUP is characterized by the classic triad of abrupt abdominal discomfort, tachycardia, and abdominal tenderness. “I hardly believe that anybody can fail in establishing a diagnosis,” Edward Crisp mentioned in 1843 [2].

Young age-group distribution is commonly seen in the developing world, which is mostly predisposed by smoking. With the advanced age in developed countries, these patients tend to be elderly with multiple comorbidities and associated use of NSAIDs, Helicobacter pylori, physiological stress, corticosteroids, and previous history of PUD are risks factors for PUP [3, 4].

Mortality risk was associated with age more than 60 years, shock (systolic pressure < 90 mmHg) at presentation, and delayed presentation (more than 24 h before surgery). Early diagnosis, prompt resuscitation, and urgent surgical intervention are essential to improve outcomes [5].

According to the diagnostic value of radiological investigation, 75% of patients with perforated peptic ulcer free air under the diaphragm were detected on erect chest/abdominal X-ray.

In comparison with a computed tomography scan which reveals superior diagnostic accuracy of 98%, a CT scan can help to distinguish other mimicking differential diagnoses of the acute abdomen like acute pancreatitis that would not require surgical intervention; the utility of this CT scan is justified when the clinical presentation is not specific to upper gastrointestinal pathology or malignancy is suspected and patients’ hemodynamic is not deranged [6].

Exploratory laparotomy and omental patch repair remain the gold standard. Laparoscopic surgery should be preserved in the early presentation of disease and diminished associated complications. Definitive anti-ulcer surgery is significantly associated with fatal outcomes in these patients, while it increases the length of the operation, exposes the patient to prolonged anesthetic time, and increases the chance of postoperative complications. Gastrectomy is recommended in patients with a large or malignant ulcer [7, 8].

The present study aimed to explore the wide range of clinical presentations, associated risk factors, complications, and surgical management among patients with perforated peptic ulcer. There has been no previous research related to this topic in the country, and this research will be a foundational study in the field.

## Methods study

### Design and study area

A 5-year retrospective observational study on the clinical presentation and surgical management of perforated peptic ulcer is carried out in a tertiary hospital in Mogadishu, Somalia, Department of General Surgery, from January 2017 to December 2021.

Mogadishu Somali Turkey *Recep Tayyip Erdoğan* Training and Research Hospital is one of the country's main referral hospitals, located in Mogadishu, the capital city of Somalia. It has a bed capacity of 250, 7 operating rooms, 28 intensive care units, 28 dialysis machines, and 300 dialysis beds, as well as a radiology department with diagnostic facilities (digital X-rays, ultrasounds, CT scanner, 1.5 T MRI, fluoroscopy) capable of performing many interventional procedures, such as ultrasound-guided and CT-guided percutaneous drainages, and serves people seeking health care from all over the country. It is also a teaching and consulting hospital that offers residency programs in 19 different specialties.

The patients were identified and extracted from hospital electronic medical records; 51 patients operated on perforated peptic ulcer for emergency surgery were reviewed, where patient clinical information including patient demographic characteristics, diagnoses, investigative (laboratory and radiological) workup, performed surgical procedure, complications, hospital stay, pathology results, peritoneal fluid culture and sensitivity, and mortality was reviewed.

A comprehensive history and physical examination were performed followed by blood investigations (e.g., CBC, liver and renal function test, electrolytes, viral markers, and blood group). Radiological investigations like X-ray abdomen erect, chest X-ray and, if necessary, an abdominal CT were performed in patients on the suspicion of diagnosis of perforated PUD.

The diagnosis of perforated peptic ulcer was confirmed with laparotomy and simple closure of the perforation by primary closure of the defect by 2.0 vicryl, and then application of the omental flap (modified Graham patch repair/omentopexy) was done.

Intraoperatively, copious saline irrigation was done and all gastric ulcers were taken a biopsy of the border of the ulcer in a systematical manner, and peritoneal fluid samples were taken for culture and sensitivity analysis.

We included all patients undergoing operations with an intraoperative confirmed diagnosis of perforated peptic ulcer at the general surgery department.

Those who operated in another hospital and were later referred to our hospital and those who operated in the pediatric surgery department were excluded from the study.

For operated patients, a 3–6-month follow-up evaluation was performed on an outpatient basis depending on their compliance.

### Data analysis

We used the Stata version 15 (StataCorp, College Station, TX) statistical program to perform statistical data analysis. Data were presented in proportions and frequency tables for categorical variables. To summarize the data for continuous variables, we utilized ranges, medians, and inter-quartile ranges (IQRs). We computed P values for categorical variables using the odds ratio (OR) and its 95% confidence interval (CI). We determined the variables associated with the outcome using logistic regression, and to adjust for confounding variables, we used multivariate logistic regression and direct standardization techniques. The significance was defined as a P value of 0.05 or less.

## Results

### Sociodemographic characteristics

Fifty-one patients underwent an emergency operation for perforated peptic ulcer during the study period. The sociodemographic distribution of patients was 45 (88.2%) males and 6 (11.8%) females, giving a male-to-female ratio of 7.5:1.

According to the age-group of the study population, they ranged from 18 to 70 years, with a mean age of 35.5 ± 16.8 years. The peak frequency was in the third decade (20–30 years).

### Clinical presentation and risk factors

The most common presenting symptoms were sudden onset of severe epigastric pain in 42 (82.4%), abdominal tenderness in 41 (80.4%), abdominal distention in 36 (70.6%), and vomiting in 31 (60.8), clinical signs of peritonitis were seen in 38 (74.5%), and < 90 mmHg systolic pressure was observed in 15 (29.4%) patients (Table 1).

**Table 1:** Clinical presentation

Clinical presentation	Frequency	Percentage
Severe abdominal pain	42	82.4
Abdominal tenderness	41	80.4
Abdominal distention	36	70.6
Vomiting	31	60.8
Classical signs of peritonitis	38	74.5
Shock on admission (SBP < 90 mmHg)	15	29.4

Two-third of the patients 34 (66.7%) were aged < 40 years; a positive previous history of peptic dyspepsia disease was noted in nearly half of the patients 25 (49%). Twenty-three patients (45.1%) had a positive history of ingestion of painkillers including nonsteroidal anti-inflammatory drugs (NSAIDS). Twenty-four patients (47.1%) had a history of cigarette smoking (Table 3).

The duration of symptoms ranged from 1 to 10 days with a mean duration of 3.8 ± 1.9 days; the median was 4 days.

The majority of patients 22 (43.1%) presented after 2–3 days of onset of symptoms. More than one-third of patients 19 (37.3%) presented of onset of symptoms between 4 and 5 days, 7 (13.7%) presented beyond 5 days after symptoms started, and only 3 (5.8%) presented within 24 h of onset symptoms.

### Diagnosis and surgical intervention

The total number of patients who performed chest/erect abdominal X-ray was 43; among these, air under diaphragm was detected in 27 (62.8%) patients. And 45 patients performed abdominal CT scan, and perforation signs were observed in 44 (97.7%) patients. Benign tissue inflammation was seen in performed pathological samples.

Modified Graham patch repair was done in nearly all the cases, and only two cases of sealed perforation underwent peritoneal lavage. Two patients were reoperated because of the failed primary repair, and in 4 patients with > 5 cm intra-abdominal abscess image-guided percutaneous drainage was performed, and in the rest antibiotic therapy was performed according to peritoneal fluid culture and sensitivity results.

**Fig. 1:**
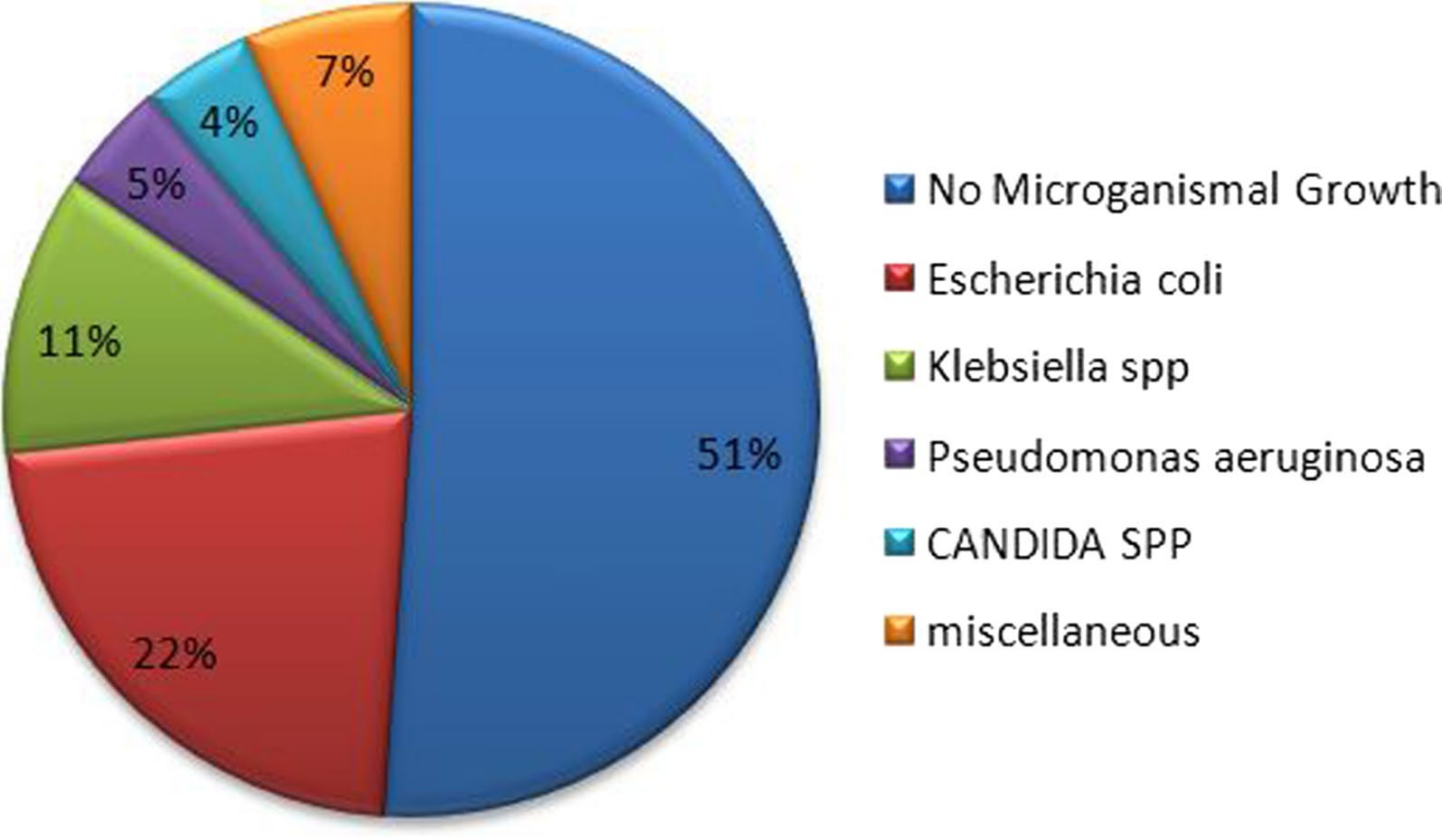
Microorganism detected in culture fluid analysis of peritoneal fluid

In half (51%) of the patients with peritoneal fluid culture, no microorganism growth was seen. The most common microorganism isolated was *E. coli* 22% and Klebsiella 11%. The others are rare microorganisms (pseudomonas, Staphylococcus aureus, and Candida spp.) (Fig. 1).

### Hospitalization duration and complications

A hospital stay of the patients ranged from minimum of 4 days to a maximum of 20 days; the mean average admission day was 8.7 ± 3.4 days.

The most common complication seen in these patients is pleural effusion which accounts for 29 (56.9%); the second foremost complication noticed in this study was acute renal failure which is about half (23 (45%)) of the patients. Surgical site infection was noted in nearly one-third (16 (31.4%)) of the patients, three patients (5.9%) developed an incisional hernia after 1 year of primary surgery, and only two patients encountered repair site leak (Table 2).

**Table 2:** Complications associated with perforated peptic ulcer

Complication	Frequency	Percentage
Atelectasis/pleural effusion	29	56.9
Acute renal failure	23	45.1
Surgical site infections	16	31.4
Intra-abdominal abscess	12	23.5
Incisional hernia	3	5.9
Leak	2	3.9

Pleural effusion was significantly high in those patients with a delayed presentation, i.e., 20 out of 29 patients came beyond 72 h of the onset of symptoms (P value 0.013).

Patients who presented perforated peptic ulcer within 24 h of initiation of symptoms were free from complications (Table 3).

**Table 3:** Associated risk factors and their frequencies

Associated risk factors	Frequency	Percentage
Age < 40	34	66.7
Cigarette smoking	24	47.1
Use of NSAIDS	23	45.1
Previous history of peptic dyspepsia	25	49

Four patients died giving a mortality rate of 7.8%, as mentioned in Table 4.

**Table 4:** Relationship between duration of perforation and postoperative complication

Duration of perforation in days	Complications						
	ARF	Atelectasis/pleural effusion	SSI	Intra-abdominal abscess	Leak	Incisional hernia	Death
1	0	0	0	0	0	0	0
2–3	9	9	6	7	0	1	1
4–5	8	14	7	9	1	1	3
> 5	6	6	3	1	1	1	1
*P* value	0.061	0.013	0.5249	0.2261	0.3755	0.7615	0.5844

### Univariate and multivariate analyses of predictors of complications

Table 5 demonstrates the predictors of complications according to bivariate and multivariate logistic regression analyses. Keeping in mind that the majority of our study population was patients less than 40 years of age, complications associated with this group were statistically significant (P 0.032).

**Table 5:** Predictors of complications according to bivariate and multivariate logistic regression analyses

Predictor (independent) variable	Complication *N* (%)	No complications *N* (%)	COR 95%CI		AOR 95%CI	
			Bivariate analysis	*P* value	Multivariate analysis	*P* value
Age						
< 40	13 (38.24)	21 (61.76)	1			
≥ 40	1 (5.88)	16 (94.12)	9.9 (0.99–98.2)	0.015	25.0 (1.31–476.65	0.032
Gender						
Male	13 (28.89)	32 (71.11)	1.000			
Female	1 (16.67)	5 (83.33)	2.031	0.209	1.84 (.11–29.44)	0.665
Cigarette smoking						
Yes	4 (16.67)	20 (83.33)	1.000			
No	10 (37.04)	17 (62.96)	0.340	0.086	.22 (.04–1.21)	.083
Use of NSAIDS						
Yes	7 (30.43)	16 (69.57)	1.000			
No	7 (25.00)	21 (75.00)	1.313	0.377	3.97 (.70–22.51)	.119
Hxdyspepsia						
Yes	6 (24.00)	19 (76.00)	1.000			
No	8 (30.77)	18 (69.23)	0.711	0.202	.54 (.10–2.77)	.461
Duration of perforation						
< 48	6 (42.86)	8 (57.14)	1			
> 48	31 (83.78)	6 (16.22)	6.9 (1.50–31.48)	0.0038	16.03 (2.34–109.5)	0.005
CRP count						
< 150	9 (25.71)	26 (74.29)	1.000			
≥ 150	5 (31.25)	11 (68.75)	0.762	0.204	.89 (.18–4.27)	.893
Site of perforation						
Gastric	10 (24.39)	31 (75.61)	1.000			
Duodenal	4 (44.44)	5 (55.56)	0.403	0.087	.12 (.014–1.13)	.065
Combined	0 (0.00)	1 (100.00)				

Alongside the age-group, also delayed presentation > 48 h after onset of symptoms was linked to postoperative complications and it is statistically significant (P 0.005).

**Fig. 2:**
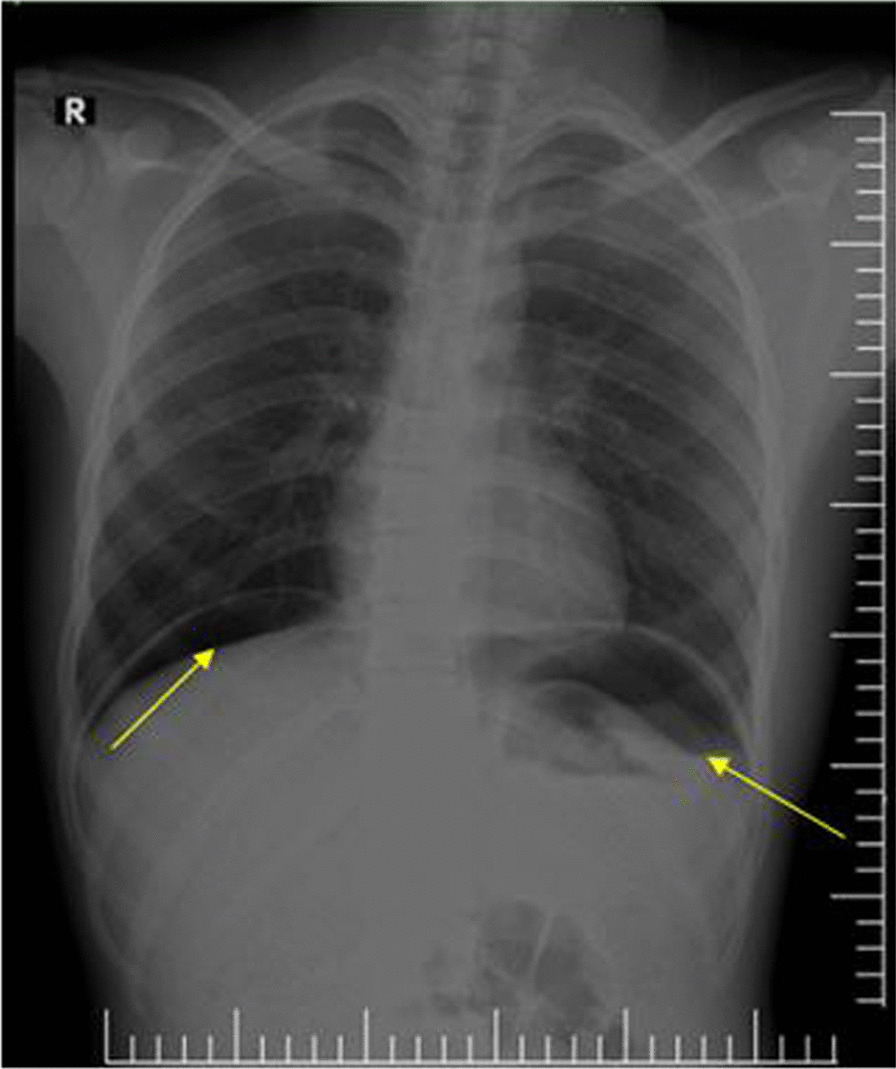
Chest X-ray showing a large volume of free sub-diaphragmatic gas with air–fluid levels under both hemidiaphragm (arrows)

**Fig. 3:**
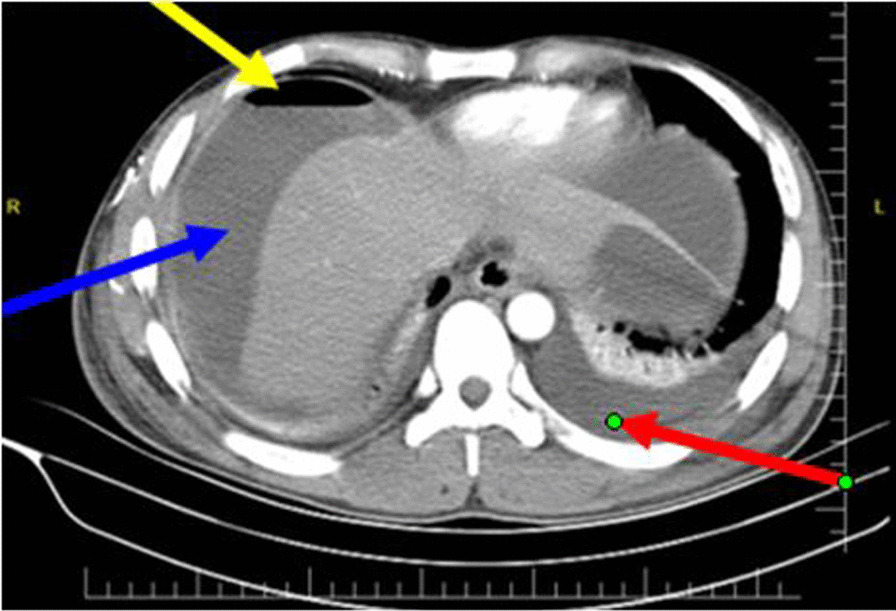
An axial abdominal CT showing free sub-diaphragmatic with air–fluid levels under right hemidiaphragm (yellow arrow), extensive free intraperitoneal fluid (blue arrow), and left pleural effusion (red arrow)

**Fig. 4:**
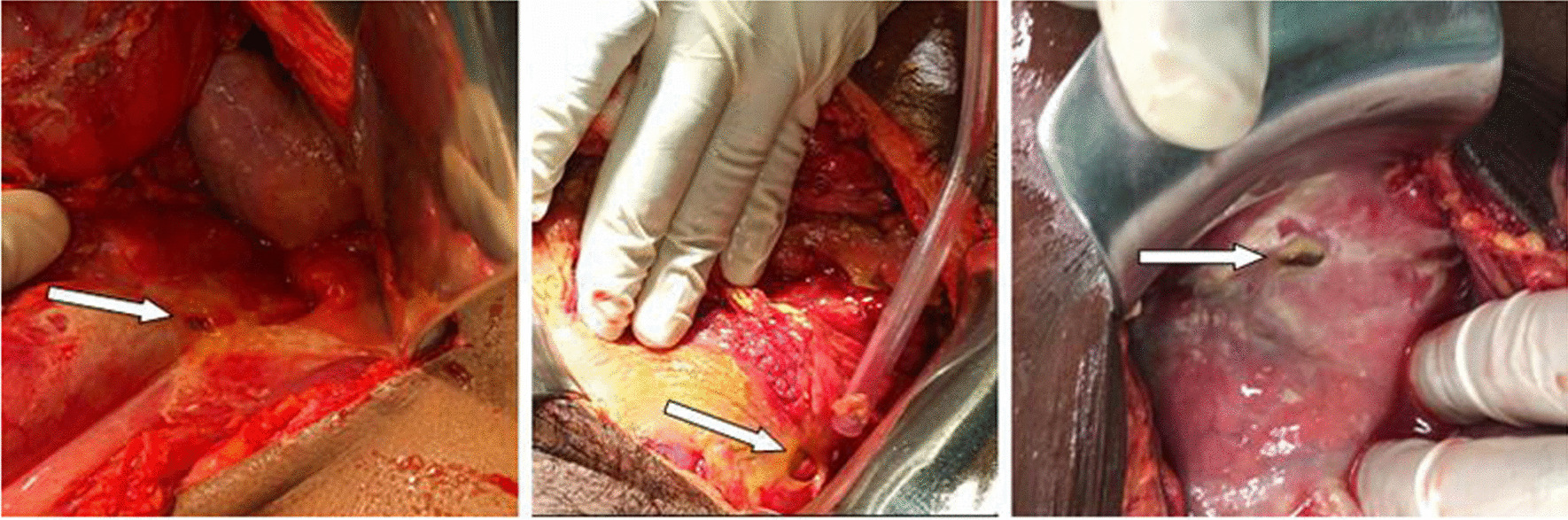
Intraoperative: perforated peptic ulcer on the anterior wall of the pyloric region in three different patients (arrows)

Higher CRP levels were attributed to the progressive inflammation and advanced peritonitis; in the present study, 68% of patients with CRP levels above 150 mg/L developed complications, but it is not statistically significant. In parallel with other examined factors, cigarette smoking, NSAID use, Hxdyspepsia, and perforation site were all found to have a non-statistically significant association with complications (Figs. 2, 3, and 4).

## Discussion

Despite the fact that perforated peptic ulcer disease is a common surgical emergency and that eradication of Helicobacter pylori has resulted in a vast decline in peptic ulcer prevalence, the number of patients requiring surgery has remained relatively constant [9].

The distribution of perforated peptic ulcer is common in the young age-group in the third decades of life; similar studies in Africa and around the globe demonstrated comparable results [8, 10, 11].

Our study demonstrated a male predominance of perforated peptic ulcer, where the majority of the patients were 45 (88.2%) males, giving a male-to-female ratio of 7.5:1 related to other studies where the male/female ratio ranged in 1.3:1 in Tanzania, 3.3:1 in Nigeria, 6.5:1 in Ethiopia, 4:1 in Singapore, and 6.8:1 in Saudi Arabia, respectively [8, 9, 12–14].

This study revealed enormous patients with delayed presentation of the disease; this is linked to most patients arriving from remote areas where proper facilities of health care and health education are not available and the patient might come to the hospital in an advanced stage of the disease; related studies agreed these findings [15, 16].

Adoption of national Essential Package of Health Services (EPHS) to all the regions of the country to have access in emergency care facility, as well as training the care providers for early identification of common signs and symptoms of abdominal emergencies, might promote preventing the late presentation of the disease.

The mean average of hospital admission days was 8.7. Long-term hospital stay was discovered as long as 20 days which correlates with late presentation of cases superimposed by consequence complications related to perforated peptic ulcer.

The comparable outcome was documented in various studies in the region and around the globe, concluding that hospital stay and related complications of PUP including death increased in delayed (> 24 h) presentation of the disease [17–19].

Positive previous history of peptic dyspepsia was observed in nearly half (25 (49%)) of the patients with peptic ulcer, this might explain the inaccessibility of good health facilities, and most of them may encounter non-professional health workers and traditional healers in many parts of the regions, and patient will seek proper health care facility when condition gets worse and patient develops severe abdominal pain and peritonism. This finding is common in developing countries as several reports stated [8, 10, 13].

The two most common microorganisms isolated were *E. coli* 22% and Klebsiella 11%; a comparable result was found in a study done in India [20].

Patients with age less than 40 years and delayed presentation of disease were found positive predictors of complications with statistically significant according to bivariate and multivariate logistic regression analyses with a p value of 0.032 and 0.005, respectively. Both factors observed a positive association with postoperative complications. This is particularly noteworthy in developing countries, including Africa, where patients often present late with severe generalized peritonitis, which correlates with a similar study in Tanzania and other parts of the world [8, 21].

The patient with a perforated peptic ulcer and sepsis should be evaluated and identified as early as possible to avoid subsequent organ failure and death [1].

There was no significant association between complications and other above-examined factors.

The mortality rate of 7.8% of patients with perforated peptic ulcer was shown in our study; this substantial rate was worldwide reported in numerous studies as 15.5, 14%, 16.7%, and 27% [19, 22–24].

This study has some limitations since it is a retrospective cross-sectional study with a single-center-based design. That might limit the generalizability of the findings.

## Conclusion

Perforated peptic ulcer is still a common surgical emergency problem in our country predominantly affecting young males and is associated with substantial morbidity and mortality in delayed disease presentation. Early diagnosis, immediate resuscitation, and urgent surgical intervention are warranted.

Simple primary repair with application omental flap (modified Graham patch repair) is the most appropriate and effective surgical approach in the management of perforated peptic ulcer.

It increases the length of the operation, exposes the patient to prolonged anesthetic time, and increases the chance of postoperative complications.

This is particularly noteworthy in developing countries, including Africa, where patients often present late with severe generalized peritonitis.

Implementation of endoscopic assessment in chronic and intractable dyspepsia will improve the early detection and management of non-complicated peptic ulcer disease.

We suggest further research, preferable to a multicenter study, to determine the epidemiology, associated risk factors, and prognostic factors of the disease in our context, and to conduct health awareness related to complications over-the-counter drugs self-medication and bad habit including smoking and to improve health-seeking behaviors of society.

## Data Availability

The data that support the findings of this study are available in Mogadishu Somali Turkey, Recep Tayyip Erdogan Training and Research Hospital information system. Data are, however, allowed to the authors upon reasonable request and with permission of the education and research committee.

## Abbreviations

PUD: Peptic ulcer disease
PUP: Peptic ulcer perforation
ARF: Acute renal failure
NSAIDs: Nonsteroidal anti-inflammatory drugs
EPHS: Essential Package of Health Services

## References

[1] Tarasconi A, Coccolini F, Biffl WL (2020). Perforated and bleeding peptic ulcer: WSES guidelines. World J Emerg Surg.

[2] Lau WY, Leow CK (1997). History of perforated duodenal and gastric ulcers. World J Surg.

[3] Xie X, Ren K, Zhou Z (2022). The global, regional and national burden of peptic ulcer disease from 1990 to 2019: a population-based study. BMC Gastroenterol.

[4] Vergara M, Catalán M, Gisbert JP, Calvet X (2005). Meta-analysis: role of Helicobacter pylori eradication in the prevention of peptic ulcer in NSAID users. Aliment Pharmacol Ther.

[5] Chung KT, Shelat VG (2017). Perforated peptic ulcer—an update. World J Gastrointest Surg.

[6] Grassi R, Romano S, Pinto A, Romano L (2004). Gastro-duodenal perforations: conventional plain film, US and CT findings in 166 consecutive patients. Eur J Radiol.

[7] Kocer B, Surmeli S, Solak C, Unal B, Bozkurt B, Yildirim O, Dolapci M, Cengiz O (2007). Factors affecting mortality and morbidity in patients with peptic ulcer perforation. J Gastroenterol Hepatol.

[8] Chaiya LP, Mabula JB, Koy M, Mchembe MD, Jaka HM, Kabangila R (2011). Clinical profile and outcome of surgical treatment of perforated peptic ulcer in Northwestern Tanzania: a Tertiary Hospital experience. World J Surg.

[9] Wadaani HA (2013). Emergent laparoscopy in treatment of perforated peptic ulcer: a local experience from a tertiary centre in Saudi Arabia. World J Emerg Surg.

[10] Ugochukwu AI, Amu OC, Nzegwu MA, Dilibe UC (2013). Acute perforated peptic ulcer: on clinical experience in an urban Tertiary Hospital in south east Nigeria. Int J Surg.

[11] Yang YJ, Bang CS, Shin SP (2017). Clinical characteristics of peptic ulcer perforation in Korea. World J Gastroenterol.

[12] Ugochukwu AI (2013). Acute perforated peptic ulcer: on clinical experience in an urban Tertiary Hospital in south east Nigeria. Int J Surg.

[13] Bupicha JA, Gebresellassie HW, Alemayehu A (2020). Pattern and outcome of perforated peptic ulcer disease patient in four teaching hospitals in Addis Ababa, Ethiopia: a prospective cohort multicenter study. BMC Surg.

[14] Anbalakan K (2015). Five year experience in management of perforated peptic ulcer and validation of common mortality risk prediction models–are existing models sufficient? A retrospective cohort study. Int J Surg.

[15] Jamieson G (2000). Current status of indications for surgery in peptic ulcer disease. World J Surg.

[16] Noguiera C, Silva A, Santos J (2003). Perforated peptic ulcer: main factors of morbidity and mortality. World J Surg.

[17] Søreide K (2015). Perforated peptic ulcer. The Lancet.

[18] Imhof M (2008). Duration of survival after peptic ulcer perforation. World J Surg.

[19] Mabewa A, Seni J, Chalya PL (2015). Etiology, treatment outcome and prognostic factors among patients with secondary peritonitis at Bugando Medical Centre, Mwanza, Tanzania. World J Emerg Surg.

[20] Srivastava R, Singh RK (2018). Clinical evaluation of patient with perforation peritonitis and their peritoneal fluid analysis for culture and sensitivity. Int Surg J.

[21] Lunevicius R, Morkevicius M (2005). Management strategies, early results, benefits, and risk factors of laparoscopic repair of perforated peptic ulcer. World J Surg.

[22] Mäkelä JT, Kiviniemi H, Ohtonen P, Laitinen SO (2002). Factors that predict morbidity and mortality in patients with perforated peptic ulcers. Eur J Surg.

[23] Montalvo-Javé EE, Corres-Sillas O, Athié-Gutiérrez C (2011). Factors associated with postoperative complications and mortality in perforated peptic ulcer. Cir Cir.

[24] Blomgren L (1997). Perforated peptic ulcer: long-term results after simple closure in the elderly. World J Surg.

